# Alterations of Photoreceptor Synaptic Ribbons in the Retina of a Human Patient With Oculocutaneous Albinism Type 1 (OCA1)

**DOI:** 10.1167/iovs.66.13.14

**Published:** 2025-10-08

**Authors:** Anna Franziska Köller, Barbara Käsmann-Kellner, Fritz Benseler, Thomas Tschernig, Ursula Löw, Stephan Maxeiner, Karin Schwarz, Nils Brose, Gerd Geerling, Berthold Seitz, Frank Schmitz

**Affiliations:** 1 Institute of Anatomy and Cell Biology, Medical School, Saarland University, Homburg, Germany; 2 Department of Ophthalmology, Saarland University Medical Center, Homburg, Germany; 3 Department of Molecular Neurobiology, Max-Planck-Institute for Multidisciplinary Sciences, Göttingen, Germany; 4 Department of Ophthalmology, University Hospital Düsseldorf, Heinrich-Heine-University, Düsseldorf, Germany

**Keywords:** OCA1, tyrosinase, melanin, albinism, human retina, photoreceptor synapse, synaptic ribbon, RIBEYE

## Abstract

**Purpose:**

Albino (Tyr^c-2J^/Tyr^c-2J^) C57BL/6J mice carry a mutation in the *tyrosinase* gene and are known to display alterations of photoreceptor synaptic ribbons. In the present study, we wanted to test whether similar alterations exist in oculocutaneous albinism type 1 (OCA1), a human disease that also results from mutations in the *tyrosinase* gene.

**Methods:**

In the present study, we assessed the morphology of a human OCA1 retina in comparison to control human retinas. We analyzed the retina of a 35-year-old OCA1 patient by immunolabeling at light and electron microscopic levels, conventional transmission electron microscopy, and by genomic DNA sequencing of the *RIBEYE/CtBP2* gene in comparison to normal human controls.

**Results:**

The morphological analyses revealed an overall surprisingly normal appearance of the retina, except for the presence of strikingly abnormal photoreceptor synaptic ribbons. Synaptic ribbons are presynaptic specializations of the continuously active retinal ribbon synapses and mainly consist of the RIBEYE protein. In the OCA1 patient, photoreceptor synaptic ribbons were very small and reduced to small fragments that were either still associated with the active zone transmitter release site or floating in the cytosol. The *RIBEYE* gene appeared to be unaltered in the OCA1 patient, except for some single nucleotide polymorphisms (SNPs) that were also present in controls.

**Conclusions:**

The OCA1 patients displayed similar defects of photoreceptor synaptic ribbons as previously observed in the albinotic mice with a defect in the *tyrosinase* gene. The observed alterations of synaptic ribbons are not due to mutations in the *RIBEYE* gene but are likely indirect consequences of the deficient melanin biosynthesis in the OCA1 patient.

Defects in melanin pigmentation in different types of albinism are known to be associated with various alterations in vision and hearing in human patients.^1^^–^^7^ Alterations of the visual system in albinotic patients include defects of foveal development and foveal hypoplasia, poor visual acuity and altered visual thresholds, pathological nystagmus, abnormal crossing of retinal axons at the optic chiasm, and altered processing of visual information in the visual cortex, indicating an important role of pigmentation in the development and function of the visual system.^1^^–^^4^^,^^8^^–^^15^ However, the molecular mechanisms are largely unclear.

Interestingly, the senses of vision and hearing, which are both affected in many albinotic patients, both employ specialized chemical synapses, so-called ribbon synapses.^16^ Ribbon synapses are specialized, tonically active glutamatergic synapses that are formed in the retina by photoreceptors and retinal bipolar cells and are also found in the inner ear and the pineal gland.^16^ Ribbon synapses are characterized at the morphological level by the presence of eponymous synaptic ribbons that are associated with the active zone transmitter release sites. Photoreceptors form particularly prominent ribbon synapses in the outer plexiform layer (OPL) and possess a relatively homogenous morphology.^16^ In the inner plexiform layer (IPL), ribbon synapses are formed by many different subtypes of retinal bipolar cells that synapse onto different types of amacrine cells and retinal ganglion cells.

Synaptic ribbons are mainly composed of the RIBEYE protein,^17^^–^^19^ which consists of a unique A-domain and a B-domain that is largely identical with the transcriptional regulator protein CtBP2.^17^ CtBP2 is highly homologous to CtBP1.^20^ Deletion of RIBEYE in RIBEYE knockout mice leads to the complete loss of synaptic ribbons in the retina and inner ear.^18^^,^^21^^,^^22^

In mice, a relationship between pigmentation and photoreceptor synaptic ribbons was observed. The albino (c2J/c2J; Tyr^c-2J^/Tyr^c-2J^) C57BL/6J mice (also known as B6 albino mice) carry a mutation in the *tyrosinase* gene.^23^^,^^24^ Remarkably, these albinotic BL6 mice display alterations of photoreceptor synaptic ribbons, which are smaller in size than those in pigmented C57BL/6J mice, and show a decreased visual sensitivity.^25^ The underlying mechanisms are not completely understood. In the present study, we wanted to analyze whether similar defects also occur in human albinism with mutations in the *tyrosinase* gene. Defects in the *tyrosinase* gene cause oculocutaneous albinism type 1 (OCA1) in humans, a rare genetic disorder in which melanin biosynthesis in the skin, hair, and eyes is partly or completely abolished.^6^^,^^26^

In the present study, we analyzed the retina of an albinotic OCA1 patient with a previously diagnosed mutation in the *tyrosinase* gene for possible alterations in photoreceptor ribbon synapses. As seen in the albino (c2J/c2J; Tyr^c-2J^/Tyr^c-2J^) C57BL/6J mouse model, we observed strong defects in photoreceptor synaptic ribbons in the OCA1 patient. Most of the photoreceptor ribbon synapses lacked synaptic ribbons or exhibited strongly altered synaptic ribbons, as assessed at the ultrastructural level. These defects are not due to mutations in the *RIBEYE* gene because the *RIBEYE* gene appeared to be un-altered in the patient except for some single nucleotide polymorphisms (SNPs) that are also present in control genomic DNA from non-albinotic healthy human donors. Rather, the observed defects of synaptic ribbons are likely secondary consequences attributable to the deficient melanin biosynthesis.

## Materials and Methods

### OCA1 Patient Data and Ethical Considerations

In the present study, we performed a morphological analysis of the retina of a male white patient with OCA1 due to mutations in the *tyrosinase* gene^27^ for morphological alterations. The *tyrosinase* gene encodes for a 529-amino-acid-long protein in humans (NM_000372.1). The OCA1 patient analyzed in the present study had several mutations in the *tyrosinase* gene, including mutations that resulted in two amino acid exchanges: Val177Phe ([c.529G>T], nomenclature according to Ogino et al.^28^), predicted as probably damaging with a score of 0.998 with PolyPhen-2 (http://genetics.bwh.harvard.edu/ggi/cgi-bin/ggi2.cgi)^29^ and predicted as deleterious by PROVEAN screen^29^ (PROVEAN score = −4.738; cutoff = −2.5), and Ala201Ser [c.601G>T], predicted as probably damaging by PolyPhen-2 with a score of 0.998 and mutation predicted as neutral by PROVEAN screen (PROVEAN score = −0.132; cutoff = −2.5) of NM_000372.1), as well as a mutation that resulted in a codon frame shift /splice site mutation [c.842delA], predicted to be deleterious by PROVEAN screen (PROVEAN score = −8.604; proveanjcvi.org) affecting the carboxyterminus of the tyrosinase protein (starting from amino acid Glu281 of NM_000372.1). Particularly the frame shift /splice site mutation and the Val177Phe amino acid exchange mutation in the *tyrosinase* gene of the OCA1 patient are predicted to strongly affect the function of the tyrosinase enzyme. The patient had a history of wearing soft contact lenses that could have fostered a diagnosed severe keratitis caused by *Pseudomonas* bacteria that developed into an endophthalmitis with the need of an emergency corneal transplantation (2005) and amniotic membrane transplantation (2010). In 2010, the patient experienced complete blindness in both eyes due to a suspected bilateral ischemic lesion of both optic nerves. Subsequently, the patient suffered strongly from severe eye pain due to his corneal problems. A developing clouding of the patient's cornea prevented further clinical imaging of the eye fundus, retina optical coherence tomography (OCT) analyses, and additional clinical imaging of the retina after the year 2011. Due to the severe eye pain, the patient requested an enucleation of his left eye which was performed in 2018 when the patient was 35 years old. The retina of the enucleated left eye was subjected to morphological analyses at the light and electron microscopy levels, as described in detail below.

All medically indicated patient enucleations were performed at the Department of Ophthalmology, Saarland University Medical Center, Homburg, Germany. Further human control retinas were obtained from body donors at the Institute of Anatomy and Cell Biology (Saarland Medical Association Ethics Committee Az. 236/22) and by samples from other human eye enucleations (typically malignant melanoma of the choroid), as indicated in the respective figures. Permission to perform the study was given by the appropriate Institutional Review Board, the ethics committee of the University/Saarland Medical Association (Ethics Committee Az. Ha50/21). Informed consent for the research was obtained from the OCA1 patient and the body and eye donors. The CARE case report guidelines (https://www.care-statement.org/checklist) were followed in the study. The research followed the tenets of the Declaration of Helsinki.

### Mouse Organ Dissection: Tissue Processing and Ethical Considerations

Mouse retina was dissected from enucleated mouse eyes within 5 minutes postmortem and processed for epoxy resin embedding and immunolabeling of epoxy resin-embedded semi-thin sections exactly as previously described.^18^^,^^30^^–^^33^ All animal procedures, including animal care, anesthesia, and euthanasia, were reviewed, approved, and supervised by the local animal committee (Landesamt für Verbraucherschutz; GB 3; 66115 Saarbrücken, Germany; GB 3-2.4.1.1-K110/180-07). The authors confirm adherence to the ARVO Statement for the Use of Animals in Ophthalmic and Vision Research.

### Processing of Human Retinas

Retina samples from enucleated eyes were processed within 1.5 hours after enucleation surgery. Enucleated eyes from body donors were obtained within 24 hours after the arrival at the Institute of Anatomy and Cell Biology in Homburg (Medical School, Saarland University). Body donors typically arrive at the Institute of Anatomy and Cell Biology 1 to 2 days after the certified death of the human body donor. Retinas were isolated from the enucleated eyes after a circular incision along the equatorial plane of the eye and removal of the lens and vitreous body from the posterior eyecup. The isolated human retina in the area between the macula region of the retina in the visual axis of the eye and the optic disc in the nasal region of the retina was cut into small pieces (∼1.5 × ∼1.5 mm in size) with a scalpel blade to improve comparability between different human retina samples. Within that region we did not consider possible regional differences in the available, analyzed human retina samples. We did not analyze the macula itself. Retina pieces from the described retina regions were differentially processed (1) for immunocytochemistry on semithin epoxy resin sections, (2) for conventional transmission electron microscopy, or (3) for post-embedding immunogold electron microscopy, as described in detail below.

### Embedding of Retinas for Immunocytochemistry on Semi-Thin Epoxy Resin Sections

For immunocytochemistry, small dissected human (and mouse) retina pieces were processed for embedding into epoxy resin exactly as previously described for mouse tissue.^30^^–^^34^ Small retina pieces were flash frozen in liquid nitrogen–cooled isopentane and freeze-dried in a vacuum generated by a DUO 004B vacuum pump (Arthur-Pfeiffer Vakuumtechnik, Wetzlar, Germany). During lyophilization, the samples were kept frozen with liquid nitrogen for ∼48 hours. The samples were then equilibrated to room temperature (RT) and infiltrated for ∼48 hours with epoxy resin. Infiltration with epoxy resin was performed at 28°C for the first 12 hours in an overhead rotator at 2 rpm to promote infiltration of the samples. Afterward, infiltration with epoxy resin was continued at RT, and samples were polymerized at 60°C for ∼48 hours. Semi-thin sections were cut from the hardened tissue blocks and processed for immunolabeling, as described below.

### Immunolabeling of Semi-Thin Epoxy Resin Sections of the Human and Mouse Retina

Immunolabeling of human and mouse retinas was performed on semi-thin (0.5 µm thin) sections of the epoxy resin–embedded retina samples. Semi-thin sections were cut from the polymerized retina tissue blocks with an ultramicrotome UltraCut E (Reichert/Jung, Nussloch, Germany) using a 45° diamond knife (DiATOME, Nidau, Switzerland) at a sectioning angle of 6° and were collected on glass coverslips. The epoxy resin was removed from the semi-thin sections, and sections were processed for immunolabeling, as previously described.^30^^–^^34^ Sections were double immunolabeled with the indicated antibodies, employing overnight incubation at 4°C with the primary antibody dilutions given in Table 2. On the next day, sections were washed several times with PBS to remove unbound primary antibodies and then incubated with the appropriate fluorophore-conjugated secondary antibodies (Table 3) for 1 hour at RT. Finally, sections were washed again several times with PBS and were embedded in *N*-propyl gallate (NPG)-antifade solution, containing 1.5% (w/v) NPG in 60% glycerol in PBS as described.^17^^,^^30^^–^^34^

**Table 1.** Patient and Eye Donor Data

Patient	Age (in Years) at Eye Enucleation	Sex	Origin
OCA1 patient	35	Male	Eye enucleation
Control group 1			
Donor 2	85	Male	Eye enucleation
Donor 3	93	Male	Body donor
Donor 4	90	Male	Body donor
Donor 5	71	Male	Body donor
Control group 2			
Donor 6	66	Male	Body donor
Donor 7	62	Female	Eye enucleation

**Table 2.** Primary Antibodies

Antibody	Reference; Source	Dilution
RIBEYE (B), mouse monoclonal (2D9)	Dembla et al.^33^	1:1000 (IF)
		1:100 (IG)
RIBEYE (B), rabbit monoclonal (U2656)	Schmitz et al.^17^	1:10,000 (IF)
PSD-95, rabbit monoclonal (L667)	Irie et al.^40^	1:500 (IF)
panPMCA, mouse monoclonal (5F10)	Krizaj et al.^41^; Invitrogen (MA3-914); RRID: AB_383600	1:300 (IF)
Cav 1.4 C-term, rabbit polyclonal	Dembla et al.^34^	1:500 (IF)

IF, immunofluorescence microscopy; IG, immunogold electron microscopy.

**Table 3.** Secondary Antibodies

Antibody	Source	Dilution
Alexa fluor 488 donkey anti-mouse	Invitrogen (A32766)	1:1000 (IF)
	RRID: AB_2866493	
Alexa fluor 488 donkey anti-rabbit	Invitrogen (A32790)	1:1000 (IF)
	RRID: AB_2762833	
Alexa fluor 568 donkey anti-mouse	Invitrogen (A10037)	1:1000 (IF)
	RRID: AB_11180865	
Alexa fluor 568 donkey anti-rabbit	Invitrogen (A10042)	1:1000 (IF)
	RRID: AB_2534017	
10 nm gold goat anti-mouse IgG	Sigma-Aldrich (G7652)	1:100 (IG)
	RRID: AB_259958	

**Table 4.** Primers for Genomic PCRs Gene Area

Gene Area	Primer	Primer Sequence (5′→3′)	PCR Product Size (bp)
*CtBP2*	A forward	AGTCCCCAGTGAGCTGTTGTC	290
*CtBP2*	B reverse	GCGAAGATTTGAGTGAGGGACTA	
*RIBEYE* A-domain	C forward	TAATCCGCATGCTGAGAGAG	870
*RIBEYE* A-domain	D reverse	GGGATCATTTTACCTCCAGGAT	
*RIBEYE* A-domain	E forward	GTCTACTCCTGACTTCACCTTCT	640
*RIBEYE* A-domain	F reverse	AGAAAGGCCAGGAACTCAG	
*RIBEYE* A-domain	G forward	ATCCCATCCAAGATGGCTTAC	990
*RIBEYE* A-domain	I reverse	GGTGAGTGAGGACTTGCCAT	
*RIBEYE* A-domain	H forward	CCACCTATTCTGCCCCTGG	520
*RIBEYE* A-domain	I reverse	GGTGAGTGAGGACTTGCCAT	
*RIBEYE* A-domain	G forward	ATCCCATCCAAGATGGCTTAC	369
*RIBEYE* A-domain	J reverse	CAGAGAAGCCAAGTCACCA	
*RIBEYE* B-domain	K forward	GTGTCAGTGGCTCCCTGGAA	883
*RIBEYE* B-domain	L reverse	ACCTGTGTCATGTGAAGGCA	
*RIBEYE* B-domain	M forward	TTCCCCACTGATCTTGAGGT	520
*RIBEYE* B-domain	N reverse	AAAGAATGCTGTCCTGCCAG	
*RIBEYE* B-domain	O forward	AGCTCAGAAGCAATGGAGGT	990
*RIBEYE* B-domain	P reverse	AAGGGCCTCGAGTTCAAAGT	
*RIBEYE* B-domain	Q forward	AAGAGCTCCCTTGTGCTGAG	755
*RIBEYE* B-domain	R reverse	TGGTGCTCACAAGCTGAGAC	
*RIBEYE* B-domain	S forward	CCAGAAGTAGAAGAGCCAAG	491
*RIBEYE* B-domain	T reverse	ACACACAATAACAGAAGTTGGTC	

**Table 5.** Genotyping

PCR Genotyping Reaction	Quantity (µL)
H_2_O	20
VWR 10× Key Buffer	3
dNTP (10 mM)	2
Forward primer (10 µM)	1
Reverse primer (10 µM)	1
Taq-polymerase	2
Template	1
Total volume	30

### Immunofluorescence Microscopy (Epifluorescence Microscopy and Confocal Microscopy)

For epifluorescence microscopy, an Axiovert 200M Fluorescence Microscope (Carl Zeiss Microscopy, Oberkochen, Germany) was used and operated under the control of the AxioVision 4.8 software. Images were acquired with a 63×/1.40 NA oil objective. Confocal microscopy was performed with a Nikon A1R confocal microscope (Nikon Corporation, Tokyo, Japan), as previously described.^32^^–^^35^ Images were acquired with a 60×/1.40 NA Plan-Apo VC oil objective using Nikon NIS-Elements 3.2 software (NIS Elements AR 3.2, 64 bit).

### Determination of Synaptic Ribbon Density in the OPL

Synaptic ribbons in the OPL, which contains the photoreceptor synapses, were counted manually in a blinded manner from the respective immunolabeled retina and related to the length of the OPL that was determined with the help of the respective scale bars. Synaptic ribbon densities, i.e., synaptic ribbon number divided through the length of the OPL (in µm) in which the synaptic ribbons were counted) were calculated. Values were expressed as synaptic ribbon number per 100 µm of OPL. For the quantitative analyses of the human retinas, the area between the fovea centralis and the optic disc was used. For the quantitative analysis of the mouse retina, sections from the central region of the retina along the visual axis were used. Statistical analyses were performed as described below.

### Embedding of Human Retinas for Post-Embedding Immunogold Electron Microscopy

Retinas obtained from eye enucleation surgeries were obtained within 1.5 hours after the surgery. Immediately after the surgery, the enucleated eyes were stored on wet ice, and the retina (dissected between the macula and the optic disc) was isolated. Within the mentioned 1.5-hour period, the retina pieces were fixed in 2% freshly depolymerized paraformaldehyde (PFA) and 0.1% glutaraldehyde in PBS (∼3 hours at 4°C). After several washes with PBS, samples were treated with 0.1% tannic acid in PBS (1 hour at 4°C) and subsequently washed with PBS first and then with 50 mM maleate buffer (pH 5.0). Next, samples were treated with 2% uranyl acetate in maleate buffer (2 hours, 4°C). Afterward, samples were washed with maleate buffer and H_2_O and dehydrated in an ascending concentration series of precooled ethanol solutions (30% and 50%, equilibrated to 4°C; 70%, 80%, 90%, and 99% (2×), pre-equilibrated to −20°C; 15 minutes each). Samples were next infiltrated with increasing concentrations of LR Gold resin (LR Gold/ethanol: 1:3, 1:1, and 3:1 [v/v] mixtures; 1 hour each at −20°C) before being transferred to pure LR Gold. LR Gold infiltration was performed overnight on an overhead rotator to promote infiltration of the LR Gold resin. The next day, the LR Gold was replaced by LR Gold containing 0.1% benzil, and samples were infiltrated for ∼2 hours on an overhead rotator. Samples were polymerized under ultraviolet light for ∼2 days at −20°C.

### Post-Embedding Immunogold Labeling of Human Retinas

Ultrathin-sections were cut with a DiATOME 45° diamond knife, at a sectioning angle of 6°, from the LR Gold–embedded human retina samples. Post-embedding immunogold microscopy with the indirect method was performed exactly as previously described.^17^^,^^30^^,^^39^ Ultrathin sections were incubated with 0.5% BSA in PBS (blocking) buffer to block unspecific binding sites (45 minutes at RT). The sections were incubated overnight with the mouse monoclonal RIBEYE antibody 2D9 (4°C). After several washes with PBS, sections were incubated with goat-anti mouse immunoglobulins that were conjugated to 5-nm gold particles (G7527; Sigma-Aldrich, St. Louis, MO, USA) used at a 1:100 dilution in blocking buffer (1 hour at RT). The unbound secondary antibody was removed by several washes with PBS, and immune complexes were fixed with 2.5% glutaraldehyde (10 minutes at RT). After several washes with PBS and then with water, sections were contrasted with 2% uranyl acetate, dried after several washes with water, and analyzed with a Tecnai BioTwin 12 transmission electron microscope (Thermo Fisher Scientific, Waltham, MA, USA) (see below).

### Preparation of Human Retina Samples for Conventional Transmission Electron Microscopy

Human retina samples were fixed with a solution containing 2.5% glutaraldehyde (EM grade), 2% freshly depolymerized paraformaldehyde in PBS, pH 7.4 (overnight 4°C). After several washes with PBS and 100-mM cacodylate buffer, samples were treated with 1% OsO_4_, 1.5% K_4_[Fe(CN)_6_]·3H_2_O in 100-mM cacodylate buffer (1 hour at 4°C) and subsequently washed thoroughly first with cacodylate buffer and then with 50 mM maleate buffer (pH 5.0). Next, samples were treated with 2% uranyl acetate in maleate buffer (2 hours at 4°C). Afterward, samples were washed with maleate buffer and H_2_O and dehydrated in an ascending concentration series of ethanol solutions (30% and 50%, equilibrated to 4°C; 70%, 80%, 90%, and 99% (2×); 15 minutes each). Samples were next infiltrated with pure acetone (15 minutes at RT) and then with increasing concentrations of epoxy resin (epoxy resin/ethanol: 1:3, 1:1, and 3:1 [v/v], 1 hour each at RT) before being transferred to pure epoxy resin. Epoxy resin infiltration was performed on an overhead rotator to promote infiltration of the epoxy resin. After infiltration with epoxy resin, samples were polymerized at 60°C for ∼2 days.

### Transmission Electron Microscopy

Ultrathin sections were analyzed with the Tecnai BioTwin 12 transmission electron microscope equipped with a MegaView III digital camera (∼2.8 megapixels, 1936 × 1456 pixels; Gatan, Unterschleissheim, Germany) that was controlled by iTEM acquisition software (Olympus, Tokyo, Japan).

### Embedding of the Optic Nerve in Paraffin Resin

The optic nerve was dissected from the posterior eye cup and fixed with 4% freshly depolymerized paraformaldehyde in PBS for 24 hours at 4°C. Embedding of the optic nerve in paraffin, sectioning, and staining of paraffin sections with hematoxylin and eosin (H&E) were performed according to standard procedures.^42^^,^^43^ Images from the H&E-stained paraffin sections were acquired with a Leica DM 2500 microscope (Leica Camera, Wetzlar, Germany) equipped with a Bresser MicroCam II Full HD digital camera operated with MicroCamLab II software (Bresser; Rhede, Germany).

### Isolation and Sequencing of Human Genomic DNA

#### Isolation of Genomic DNA

Genomic DNA from the OCA1 patient and two healthy, non-albinotic human controls was isolated from ethylenediaminetetraacetic acid (EDTA)-treated donor blood. Genomic DNA isolation from donor blood was done with a Quick-DNA Miniprep Plus Kit (Zymo Research Corporation, Irvine, CA, USA) according to the manufacturer's instructions. In brief, 200 µL EDTA/blood plus 200 µL Biofluid & Cell Buffer (Zymo Research) and 20 µL Proteinase K were incubated at 55°C (overnight [ON]) in a shaker with mild agitation. Afterward, 420 µL Genomic Binding Buffer (Zymo Research) was added, and the entire mixture was added to the DNA binding column. After brief centrifugation (1 minute at 13,000 rpm in a Sorvall Heraeus #3328 rotor; Thermo Fisher Scientific), the column was subsequently washed with 400 µL DNA-Pre-Wash Buffer (Zymo Research), 700 µL g-DNA-Wash Buffer, and finally 200 µL g-DNA-Wash Buffer before the genomic DNA was eluted with 20 µL elution buffer. Eluted DNA was stored at 4°C. DNA concentration was determined with a NanoDrop One spectrophotometer (Thermo Fisher Scientific).

#### Primers and Templates

The following primers given in Table 4 were used for sequencing of *RIBEYE/CtBP2* genomic DNA (Table 5). As template, we used 1 µL (typically containing 30–60 ng DNA) of the isolated genomic patient DNA or human control genomic DNA. PCR primers (Table 4) were diluted from a 100-µM stock solution and used at a final concentration of 10 µM.

#### PCR Amplification of Genomic DNA

PCR cycling conditions were as follows: 5 minutes at 94°C (initial denaturation); 20 seconds at 94°C, 30 seconds at 60°C, and 50 seconds at 72°C (40 cycles at 72°C; 10-minute final extension at 4°C). Each PCR amplification was done in triplicate, and the PCR reactions were subjected to direct sequencing. Nucleotide variants were only considered valid if they were observed in all three independent experiments excluding those based on proofreading errors during DNA amplification.

#### Direct DNA Sequencing of PCR Products

Prior to sequencing, all PCR products were purified from 1% to 1.5% agarose gels using the QIAquick Gel Extraction Kit (Qiagen, Hilden, Germany). The concentration of the isolated DNA was then determined using a NanoDrop One spectrophotometer and adjusted to 20 ng/µL. In order to obtain sequence data with a high level of accuracy for the different RIBEYE domains, the PCR fragments for the RIBEYE A-domain were designed in an overlapping format and used in different combinations. In addition, all generated PCR products were sequenced from both sites with the forward and reverse primers used to amplify the corresponding PCR fragments. The combined sequence data from both directions were used for subsequent analyses of the amplified regions. Sequencing reactions were performed using the dideoxy chain terminator cycling sequencing method in a ProFlex PCR System (Thermo Fisher Scientific). The sequencing reactions (12 µL) were prepared in 96-well plates, and any used reaction well contained 4 µL PCR product, 4 µL sequencing primer (1 pmol/µL), and 4 µL Applied Biosystems BigDye Sequencing Master Mix (Thermo Fisher Scientific). The composition of the 4-µL Master Mix for one reaction was as follows: 0.3 µL BigDye Terminator v3.1 Cycle Sequencing Kit, 1.05 µL 5× BigDye Sequencing Buffer (part of the BigDye Kit), 2.4 µL 5× Sequencing Enhancement Buffer for BigDye, and 0.25 µL H_2_O (Arium Pro Ultrapure Lab Water System; Sartorius, Göttingen, Germany). The thermal cycling profile for performing the sequencing reaction was as follows: 1 minute at 96°C (initial denaturation), 10 seconds at 96°C, 5 seconds at 50°C, 2 minutes at 60°C, and 34 cycles at 4°C. The resulting sequencing products were purified by ethanol precipitation to remove unincorporated fluorescently labeled nucleotides and analyzed by capillary electrophoresis using an Applied Biosystems 3730XL DNA Analyzer (Thermo Fisher Scientific). The obtained raw data were analyzed with Sequencing Analysis Software 7 (Life Technologies, Darmstadt, Germany) and further analyzed using the Dotmatics SnapGene software package (formerly GSL Biotech, Boston, MA, USA).

### Statistical Analyses

Statistical analyses of synaptic ribbon densities were performed with Prism 10 (GraphPad, Boston, MA, USA). Data within the three individual groups (OCA1 patient retina, retinas from donors younger than 70 years, and retinas from donors older than 70 years) were pooled. All data obtained from the human donors were normally distributed, as determined by the Shapiro–Wilk test. For individual comparisons, Student's *t*-test was applied; for the multiple comparisons of human retinas, ANOVA with Tukey post hoc correction for multiple comparisons was used.

## Results

We analyzed the retina of a male white OCA1 patient for possible morphological alterations. The enucleation of the left eye was performed in the year 2018, when the patient was 35 years old, because of severe painful keratitis/endophthalmitis of the eye. The retina of the enucleated left eye was subjected to light and electron microscopy analyses. The results of these morphological analyses are documented in the present study.

The clinical fundus examination revealed a typical image as expected for an albinotic OCA1 patient (Supplementary Fig. S1). Due to the absence of melanin pigment in the retinal pigment epithelium (RPE), not only were the vessels of the central retinal artery and central retinal vein visible but also the blood vessels of the choroid (Supplementary Fig. S1).

**Figure 1.**
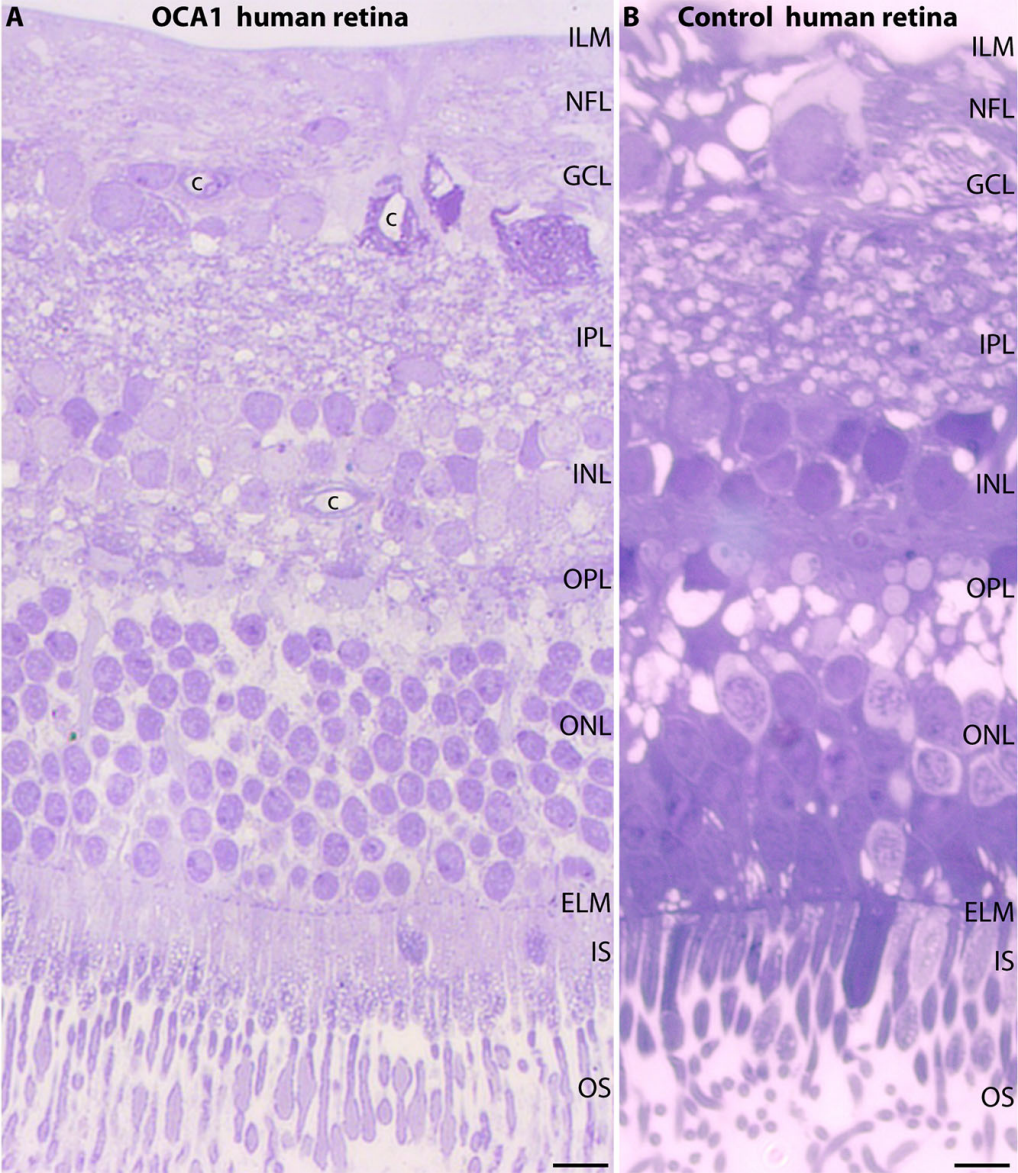
(**A**, **B**) Semi-thin epoxy resin section of the retina from the 35-year-old human OCA1 patient (**A**) and a human control retina from a non-albinotic human donor (62 years old) (**B**) stained with Richardson Blue. The retina pieces were dissected from the central nasal retina located between the macula and the optic disc. *Scale bar*: 5 µm. c, capillary; ELM, external limiting membrane; GCL, ganglion cell layer; ILM inner limiting membrane; INL, inner nuclear layer; IPL, inner plexiform layer; IS, inner segment; NFL, nerve fiber layer; ONL, outer nuclear layer; OPL, outer plexiform layer; OS, outer segment.

Standard light microscopy evaluation of the retina from the enucleated left eye of the OCA1 patient did not show obvious abnormalities despite the complete bilateral vision loss of the patient (Fig. 1). A 0.5-µm-thin (semi-thin) resin section of the patient retina (stained with Richardson Blue) revealed a well-preserved and normal-appearing retina, with well-differentiated retinal layers (Fig. 1). The retina appeared well preserved at the light microscopy level.

The photoreceptor outer segments (OSs), which are responsible for phototransduction, were clearly visible in the OCA1 patient's retina and appeared normal at the light microscopy level (Fig. 1). Furthermore, all retinal nuclear layers (i.e., the outer nuclear layer [ONL]), the inner nuclear layer (INL), and the ganglion cell layer (GCL), were well maintained (Fig. 1), without obvious alterations as compared to human retina images in standard textbooks.^44^

**Figure 2.**
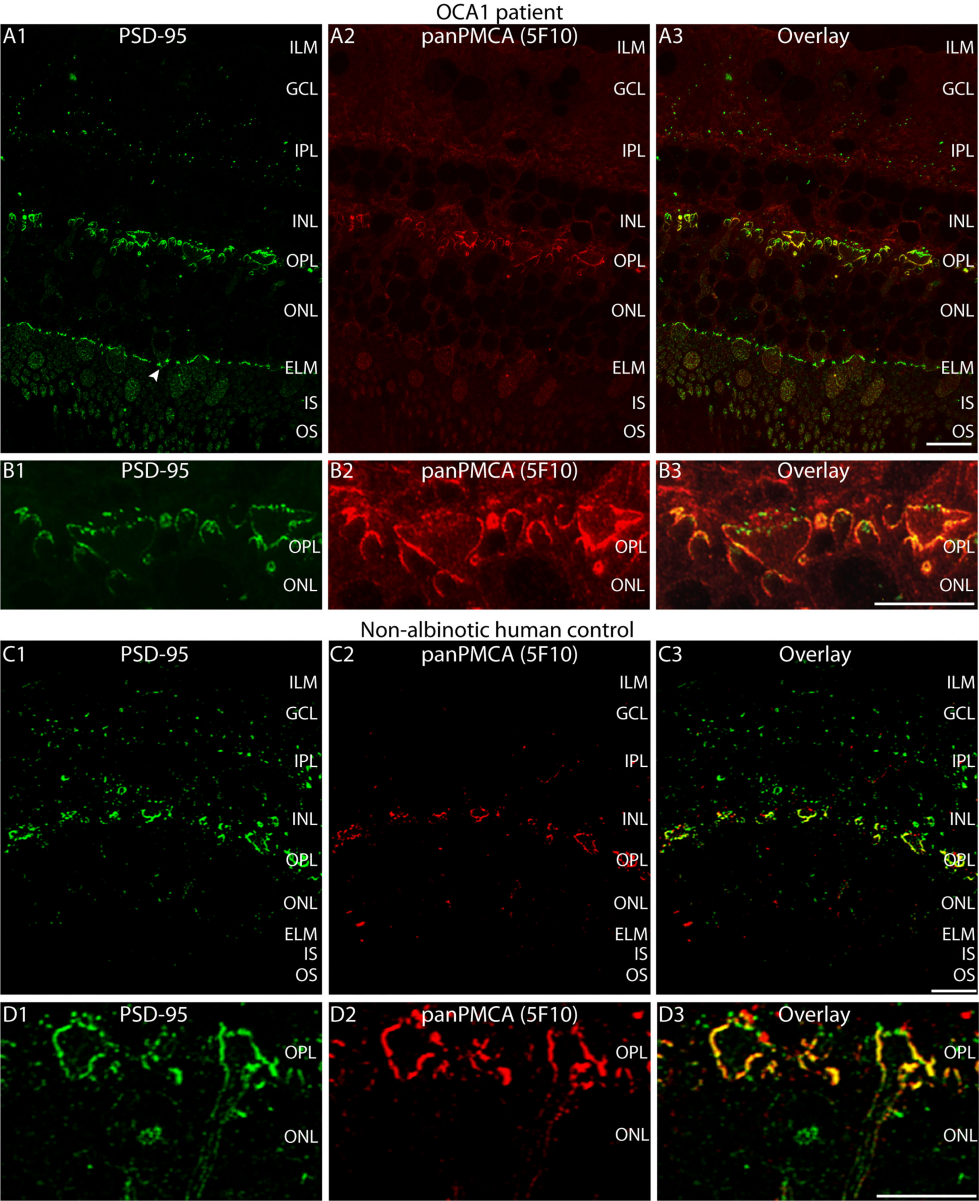
(**A–D**) Double immunolabeling of 0.5-µm-thin retina sections from the OCA1 patient (**A1**–**A3**, **B1**–**B3**) and from a non-albinotic human control donor (**C1**–**C3**, **D1**–**D3**) with rabbit polyclonal antibody against PSD-95 (L667, in *green channel*) (**A1**, **B1**, **C1**, **D1**) and mouse monoclonal antibody against PMCA (clone 5F10, in *red channel*) (**A2**, **B2**, **C2**, **D2**). Signals are merged in **A3**, **B3**, **C3**, and **D3**. *Arrowhead* in **A1** points to an unusual PSD-95 signal in the ELM of the OCA1 patient. The retina pieces were dissected from the central nasal retina located between the macula and the optic disc. Please note that the images from the immunolabeled retinas are presented in the OCT orientation (*top*, inner retinal layers; *bottom*, outer retinal layers). *Scale bar*: 5 µm.

Immunofluorescence microscopic analyses of the OCA1 patient retina performed with antibodies against postsynaptic density protein of 95 kDa (PSD-95) and a panPMCA antibody directed against the plasma membrane Ca^2+^ ATPase (PMCA) exchanger protein did not show obvious abnormalities in most regards (Figs. 2A1–A3, 2B1–B3) in comparison to a non-albinotic human control retina (Figs. 2C1–C3, 2D1–D3). PSD-95 is a postsynaptic density protein in most of the central nervous system synapses.^45^ In photoreceptor synapses, PSD-95 is a presynaptic marker that forms a subplasmalemmal network along the presynaptic plasma membrane.^45^ At this presynaptic location, PSD-95 co-localizes with PMCA.^35^

The PSD-95 signals were very similar to those previously published,^36^^,^^38^^,^^45^ showing strong immunosignals in the first synaptic layer of the retina, the OPL, which contains the photoreceptor synapses, and, to a lesser extent, immunolabeling of the second synaptic layer, the IPL. In the OPL, we observed the typical honeycomb pattern for PSD-95 that is characteristic for a protein located beneath the presynaptic plasma membrane of photoreceptor presynaptic terminals. In the IPL, the PSD-95 pattern was punctate, as is typical for a postsynaptic density protein and similar to previously published data.^36^^,^^45^ The only unusual observation we made was the additional PSD-95 immunosignals close to the external limiting membrane (ELM) in the OCA1 patient retina (Fig. 2A1) which was not present in the non-albinotic human control retina (Fig. 2C1). At the photoreceptor synapses in the OPL, the PMCA and PSD-95 immunosignals largely overlapped both in the OCA1 patient retina and in the human control retina (Figs. 2B, 2D).

**Figure 3.**
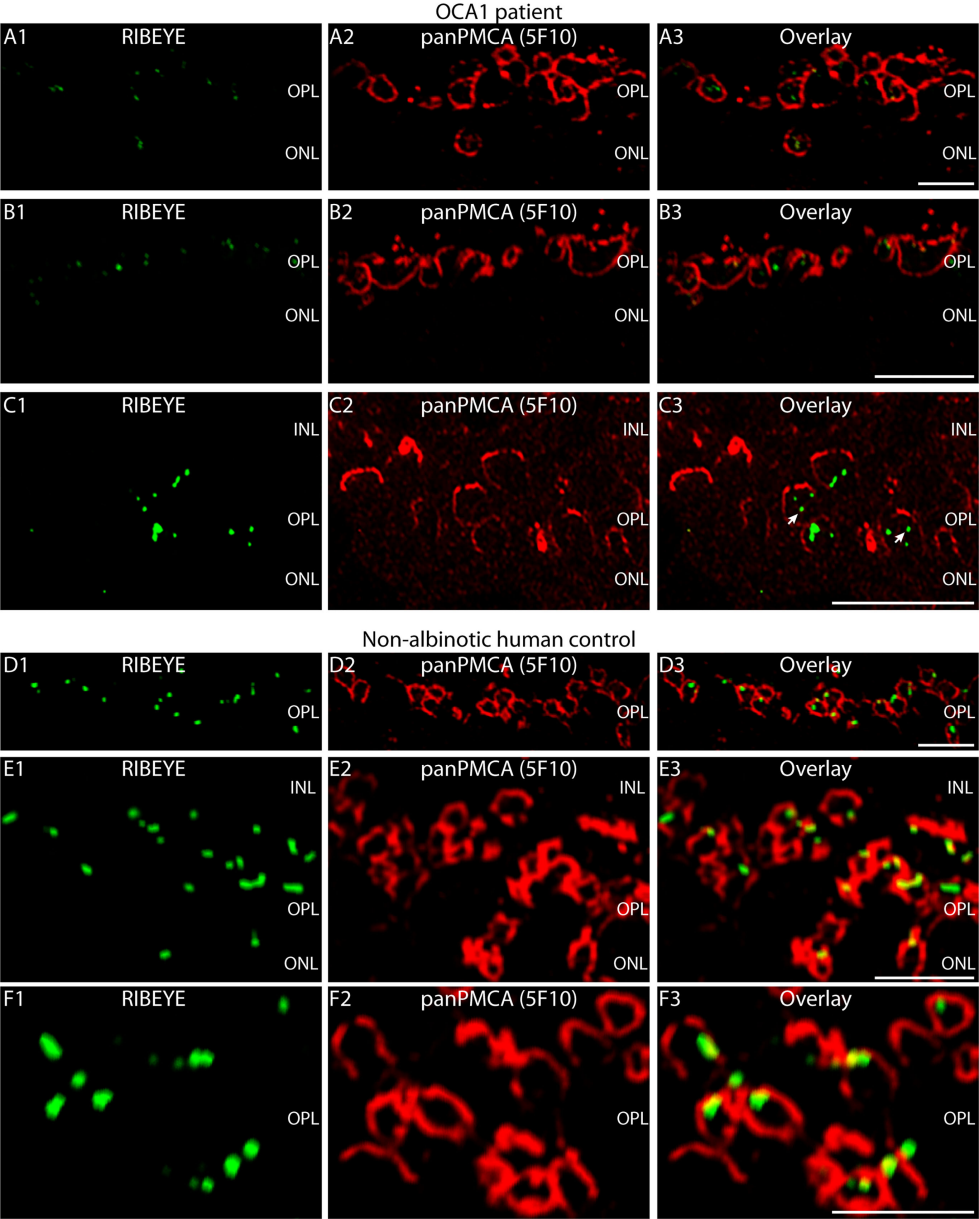
(**A–F**) Double immunostaining of 0.5 µm-thin retina sections of the OCA1 patient (**A**–**C**) and a non-albinotic control human eye donor (**D**–**F**) with rabbit polyclonal antibody against RIBEYE (U2656, in *green channel*) and mouse monoclonal antibody against panPMCA (clone 5F10, in *red channel*). Signals of the *green channels* and *red channels* are merged in **A3**, **B3**, **C3**, **D3**, **E3**, and **F3**. *Arrows* in **C3** point to “floating” synaptic ribbons: synaptic ribbons that appear to be detached from the plasma membrane of the photoreceptor presynaptic terminal that was immunolabeled with anti-panPMCA. The retina pieces were dissected from the central nasal retina located between the macula and the optic disc. Please note that the images from the immunolabeled retinas are presented in the OCT orientation (*top*, inner retinal layers; *bottom*, outer retinal layers). *Scale bar*: 5 µm.

Double immunolabeling experiments with antibodies against PMCA and RIBEYE (U2656) revealed abnormal immunosignals for RIBEYE in photoreceptor synapses of the OCA1 patient retina (Figs. 3A–C) in comparison to non-albinotic human control retina (Figs. 3D–F). The RIBEYE immunosignals were sparse in the OPL of the OCA1 patient, and only a subset of PMCA-labeled photoreceptor presynaptic terminals contained a synaptic ribbon (Figs. 3A–C). Furthermore, many RIBEYE immunosignals were spatially segregated from the PMCA-labeled presynaptic plasma membrane and appeared to be located inside the presynaptic terminal, indicating major synaptic ribbon abnormalities in photoreceptor synapses of the OCA1 patient (Figs. 3A–3C). In contrast, control photoreceptor synapses showed synaptic ribbons close to the PMCA-labeled presynaptic plasma membrane (Figs. 3D–F).

**Figure 4.**
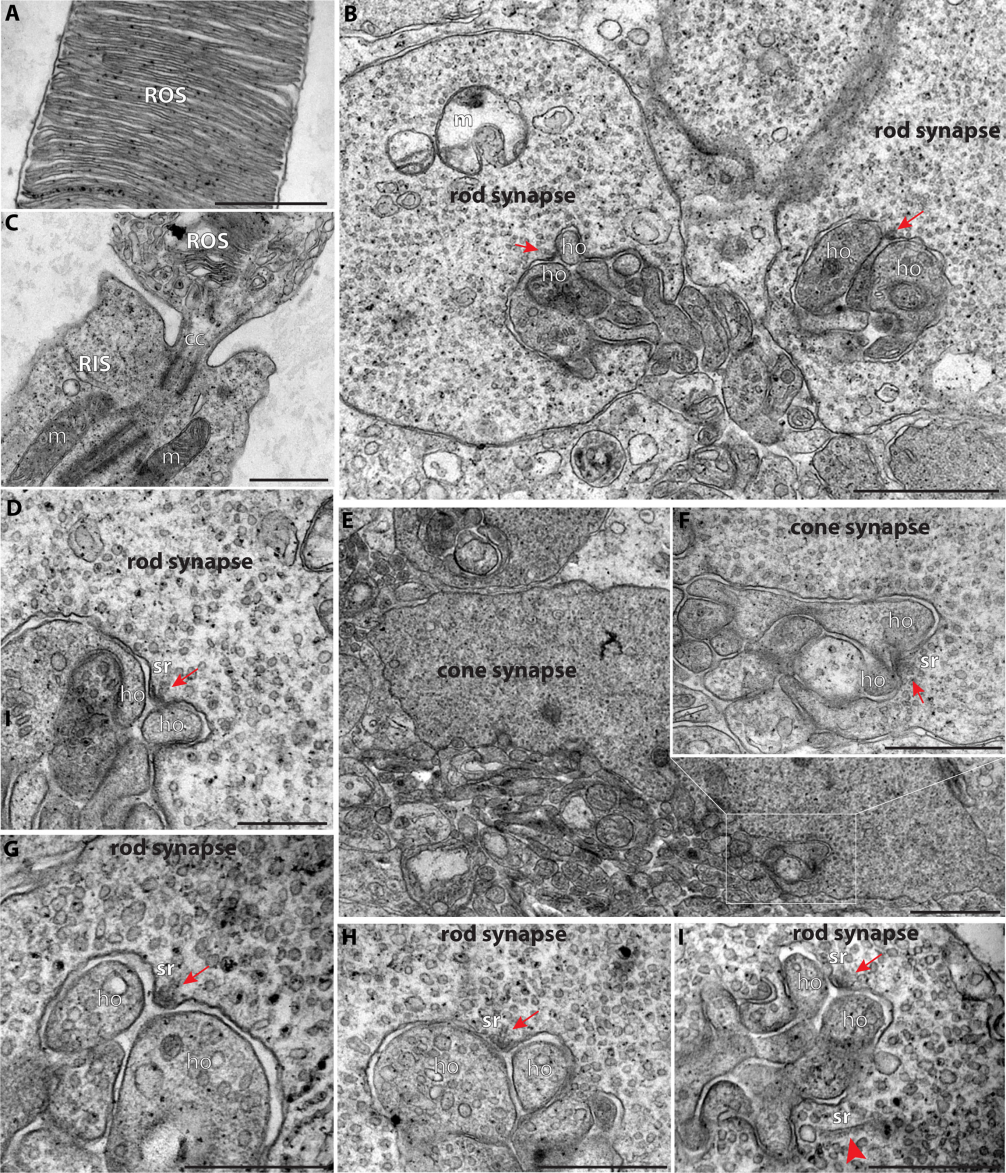
(**A–I**) Ultrastructure of the retina from the OCA1 patient, as analyzed by conventional transmission electron microscopy. (**A**) Rod outer segment. (**C**) Rod outer segment and rod inner segment linked by the connecting cilium. (**B**, **D**–**I**) Photoreceptor synapses in the outer plexiform layer. *Arrows* in **B**, **D**, **F**, **G**, **H**, and **I** point to tiny synaptic ribbon remnants at the active zone. *Arrowhead* in **I** denotes a fragmented synaptic ribbon floating in the cytosol. *Scale bars*: 500 nm. ROS, rod outer segment; RIS, rod inner segment; sr, synaptic ribbon; cc, connecting cilium; m, mitochondrion; ho, dendritic tips of horizontal cells.

**Figure 5.**
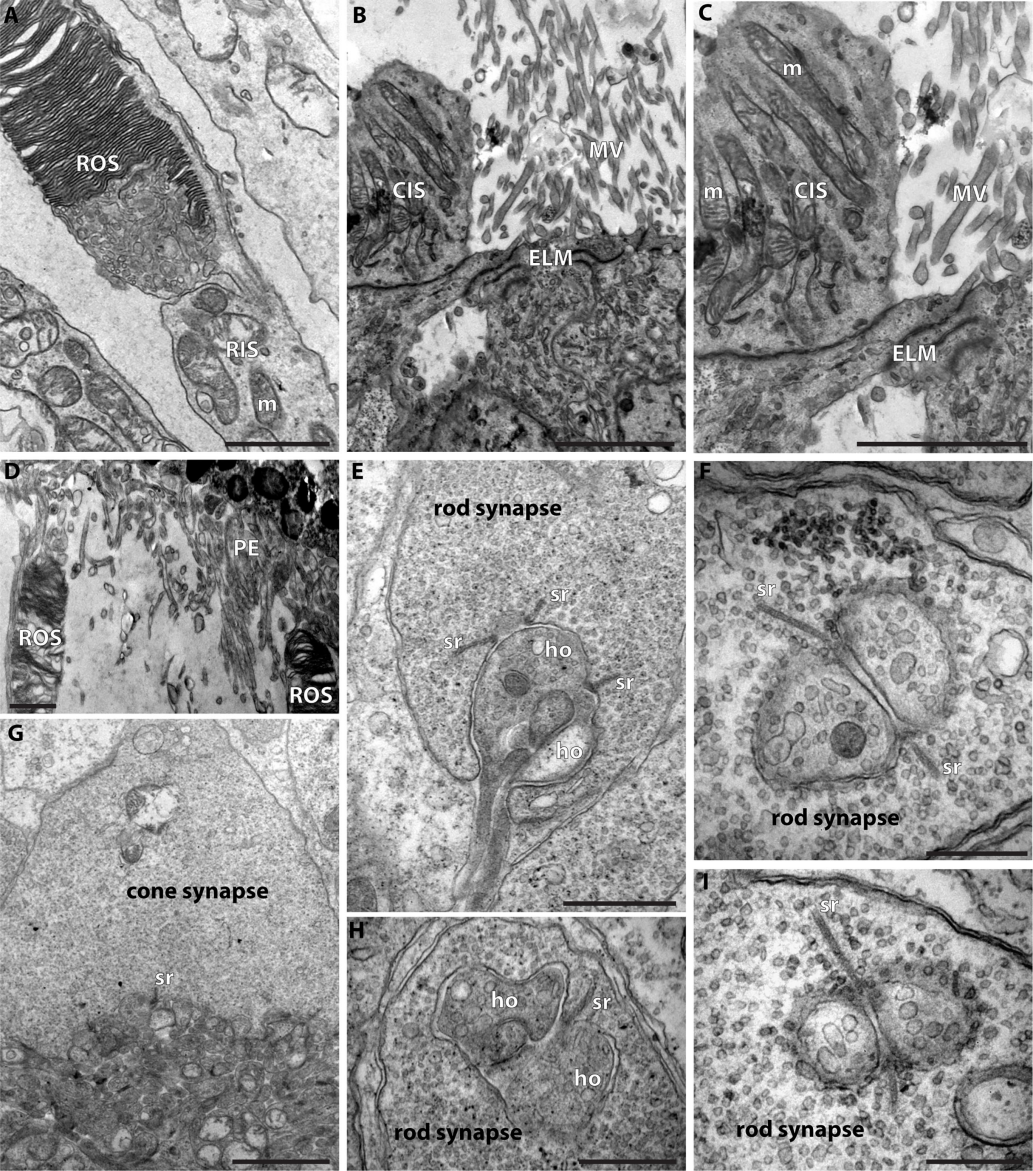
Transmission electron microscopy of a control retina (62-year-old donor). (**A**) Rod outer segment. (**B**, **C**) External limiting membrane. (**D**) Contact site between rod outer segments and pigment epithelium. (**E**–**I**) Photoreceptor synapses. *Scale bars*: 1 µm (**A**, **D**, **G**); 500 nm (**B**, **C**, **E**, **F**, **H**, **I**). CIS, cone inner segment; MV, microvilli of Müller glia cells; PE, pigment epithelium.

We next analyzed this apparent synaptic ribbon phenotype at the ultrastructural level by conventional transmission electron microscopy. At the ultrastructural level, photoreceptor synapses in the OPL can be easily identified by their typical localization and characteristic morphology.^16^^,^^44^^,^^46^^,^^47^ The morphology of the photoreceptor outer segments, which are responsible for phototransduction, was perfectly preserved (Figs. 4A, 4C), containing many densely packed discs and linked to the inner segment with a connecting cilium. The presynaptic terminals of rod and cone photoreceptor synapses also appeared largely normal, containing many synaptic vesicles and exhibiting the characteristic morphology with invaginations of the presynaptic terminal (Figs. 4B, 4D–I).^16^^,^^44^ A striking ultrastructural defect was the absence of synaptic ribbons from many active zones and an altered morphology of the remaining synaptic ribbons (Figs. 4B, 4D–I). The synaptic ribbons were either absent from the active zone or barely detectable. Higher magnification of the active zone showed that small fragments of synaptic ribbons were often still attached to the active zone. However, these remnants were much shorter than normal synaptic ribbons and were difficult to identify by conventional electron microscopy due to an atypical appearance displaying decreased electron density. In some instances, floating, detached synaptic ribbons were found in the cytosol (Fig. 4I), which could be the ultrastructural correlate for the RIBEYE-positive signals detected in the center of the presynaptic terminals using immunofluorescence microscopy (Figs. 3C1, 3C3). These ultrastructural alterations of photoreceptor synaptic ribbons were typical for the analyzed OCA1 patient and not a general feature of humans, because photoreceptor synapses of human control retinas displayed normal synaptic ribbons (Fig. 5).

**Figure 6.**
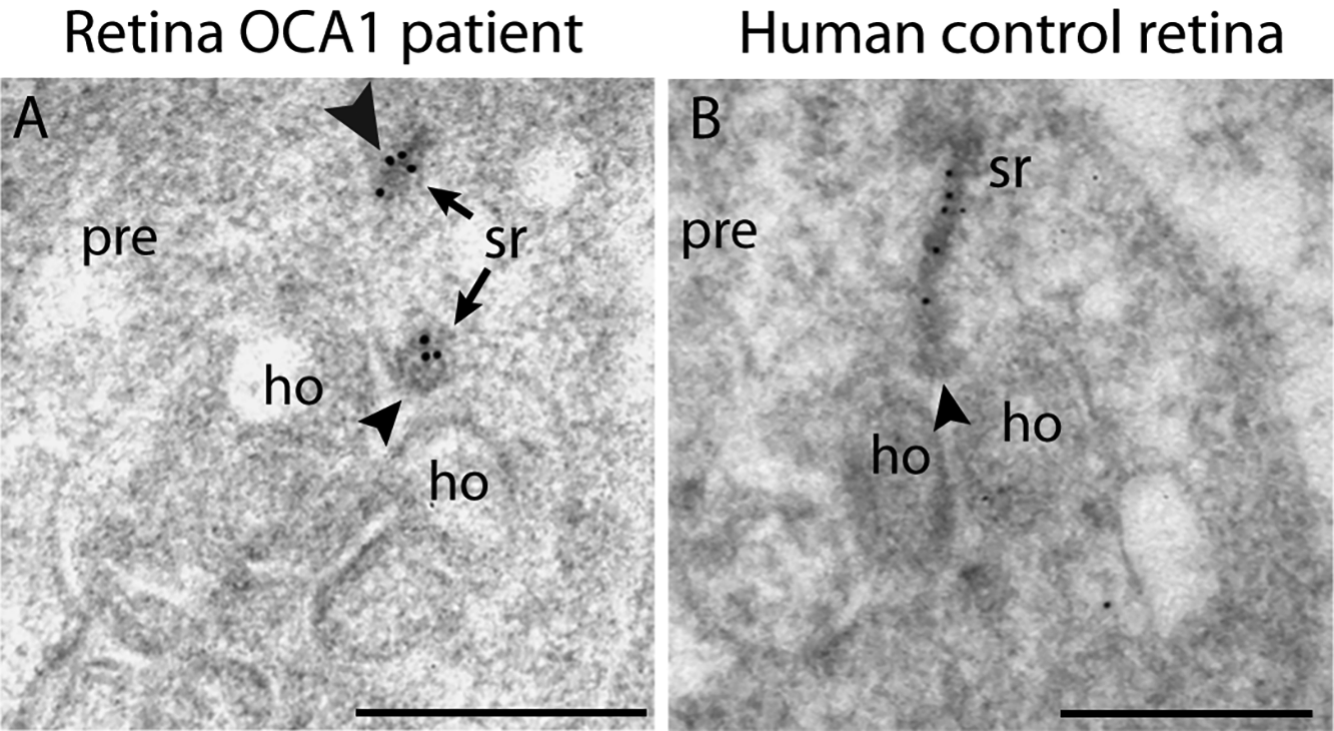
(**A**, **B**) Ultrathin LR Gold section of a rod photoreceptor synapse from the OCA1 patient (**A**) or a control human retina (**B**) immunolabeled by indirect post-embedding immunogold microscopy with antibodies against RIBEYE, the main component of synaptic ribbons. The *arrowhead* points to the active zone in which the synaptic ribbon is anchored. *Scale bars*: 300 nm; pre, presynaptic.

Post-embedding immunogold electron microscopy of the OCA1 patient samples confirmed that the active zone of rod synaptic ribbons contained only tiny RIBEYE-positive synaptic ribbon remnants. The synaptic ribbons appeared fractured with one tiny portion remaining attached to the active zone and another part of the synaptic ribbons floating in the cytosol of the presynaptic terminal (Fig. 6A, arrowhead). Synaptic ribbons of a control human retina showed no such defects (Fig. 6B).

**Figure 7.**
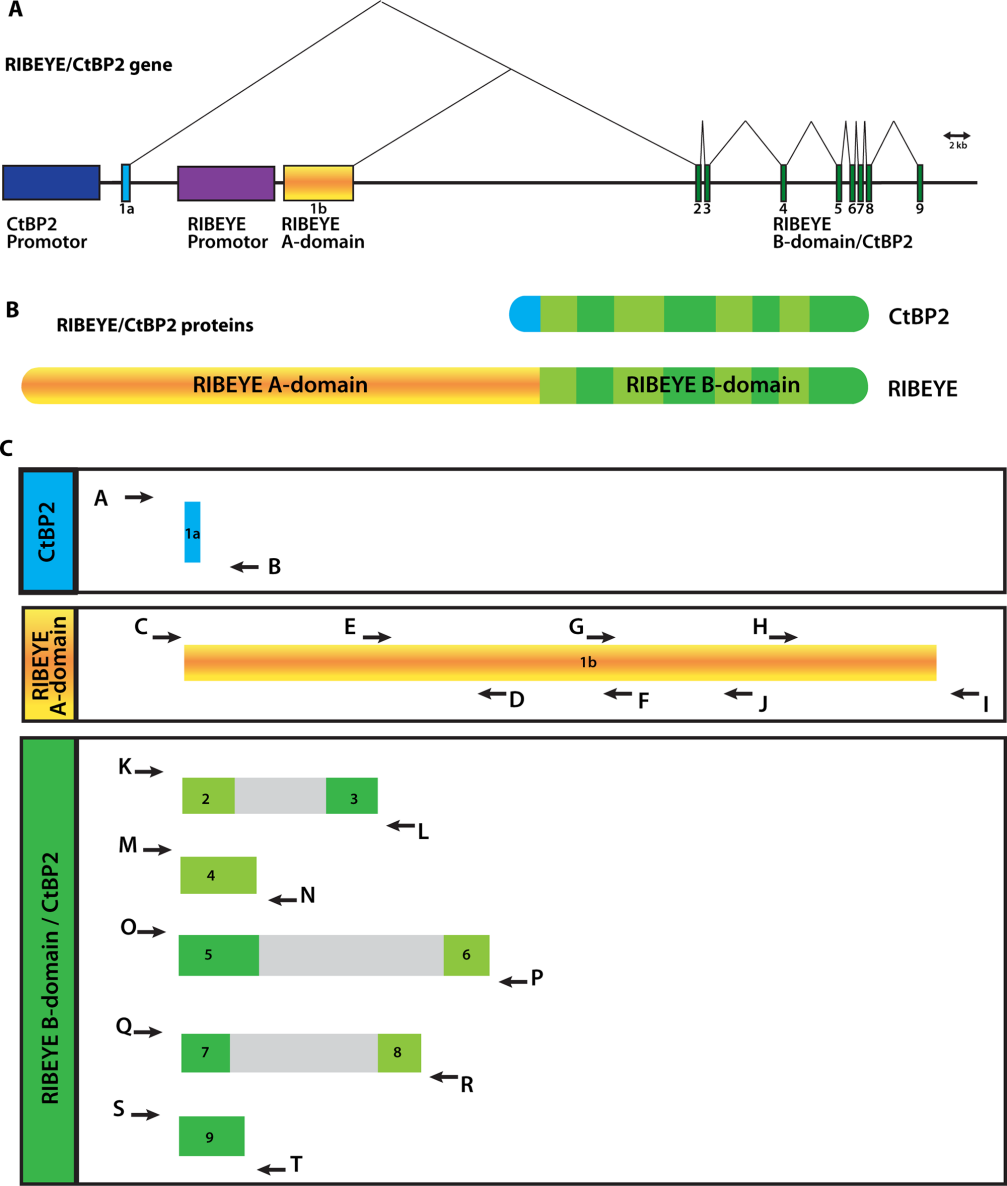
(**A**) Schematic organization of the *RIBEYE/CtBP2* gene. The CtBP2 promotor drives ubiquitous expression of the CtBP2 protein, whereas the RIBEYE promotor drives expression of RIBEYE only in ribbon synapse forming cells (drawing modified from Reference 16). Exon1a, which is unique to CtBP2, is shown in *blue*. The RIBEYE-specific exon 1b, encoding RIBEYE A-domain, is shown in *yellow*, and the eight common exons encoding RIBEYE B-domain/CtBP2 are shown in *green*. (**B**) Schematic demonstrates the proteins that are expressed from the multifunctional *RIBEYE/CtBP2* gene. (**C**) Schematic depicts the exons that have been sequenced after PCR amplification with the indicated primers. The full exons were sequenced together with the intron/exon boundaries.

Because the synaptic ribbons exhibited the only major structural defect in the retina of the OCA1 patient, we analyzed the *RIBEYE* gene of the OCA1 patient to exclude the possibility that an additional variation in the *RIBEYE* gene caused the observed synaptic ribbon alterations. RIBEYE is the major protein component of synaptic ribbons.^17^^,^^18^ The *RIBEYE* gene is a multifunctional gene that generates multiple gene products (Figs. 7A, 7B).^17^^,^^48^

**Figure 8.**
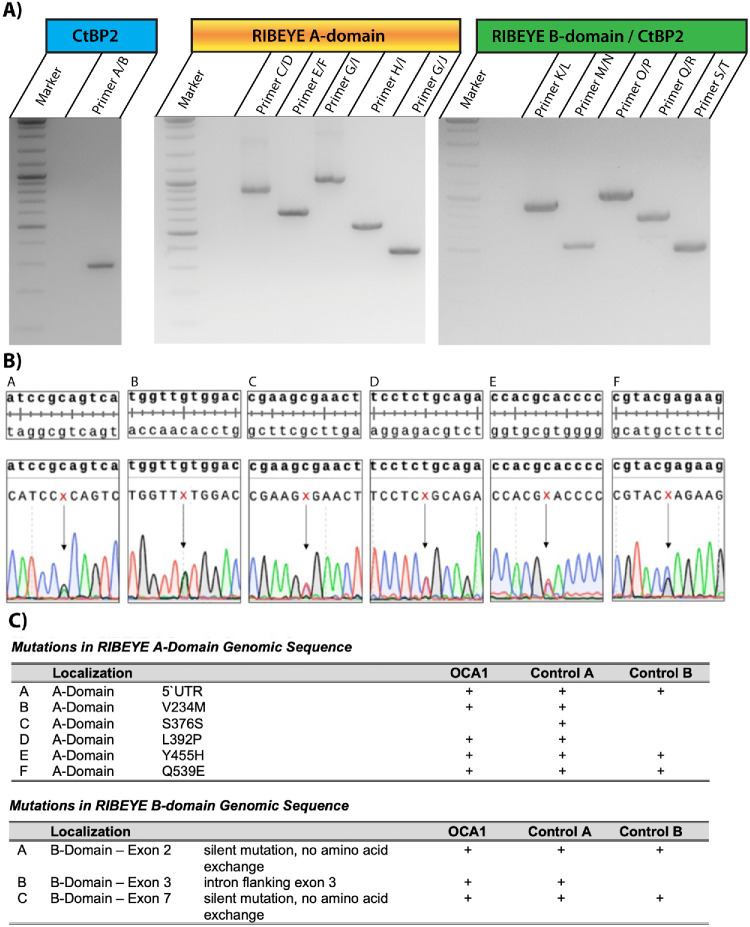
(**A**) Representative agarose gel images of the indicated PCR genotyping reactions. (**B**) Electropherograms of the depicted mutations. (**C**) Summary of mutations in the RIBEYE genomic sequences that differ from genomic database entry NC 060934.1 for human RIBEYE on chromosome 10. None of the identified mutations occurred only in the OCA1 patient but not in control genomic DNA (control A and control B) obtained from two healthy non-albinotic human donors. For the RIBEYE B-domain, we only observed silent mutations.

We sequenced all exons of the *RIBEYE* gene as shown in Figures 7A and 7C. Not unexpectedly, we found several variations (i.e., deviations from the database genomic DNA entries for *RIBEYE*) (Fig. 8). But, these are unlikely to account for the observed synaptic ribbon phenotype of the OCA1 patient as they appeared to be SNPs that are also present in control genomic DNA (Fig. 8).

**Figure 9.**
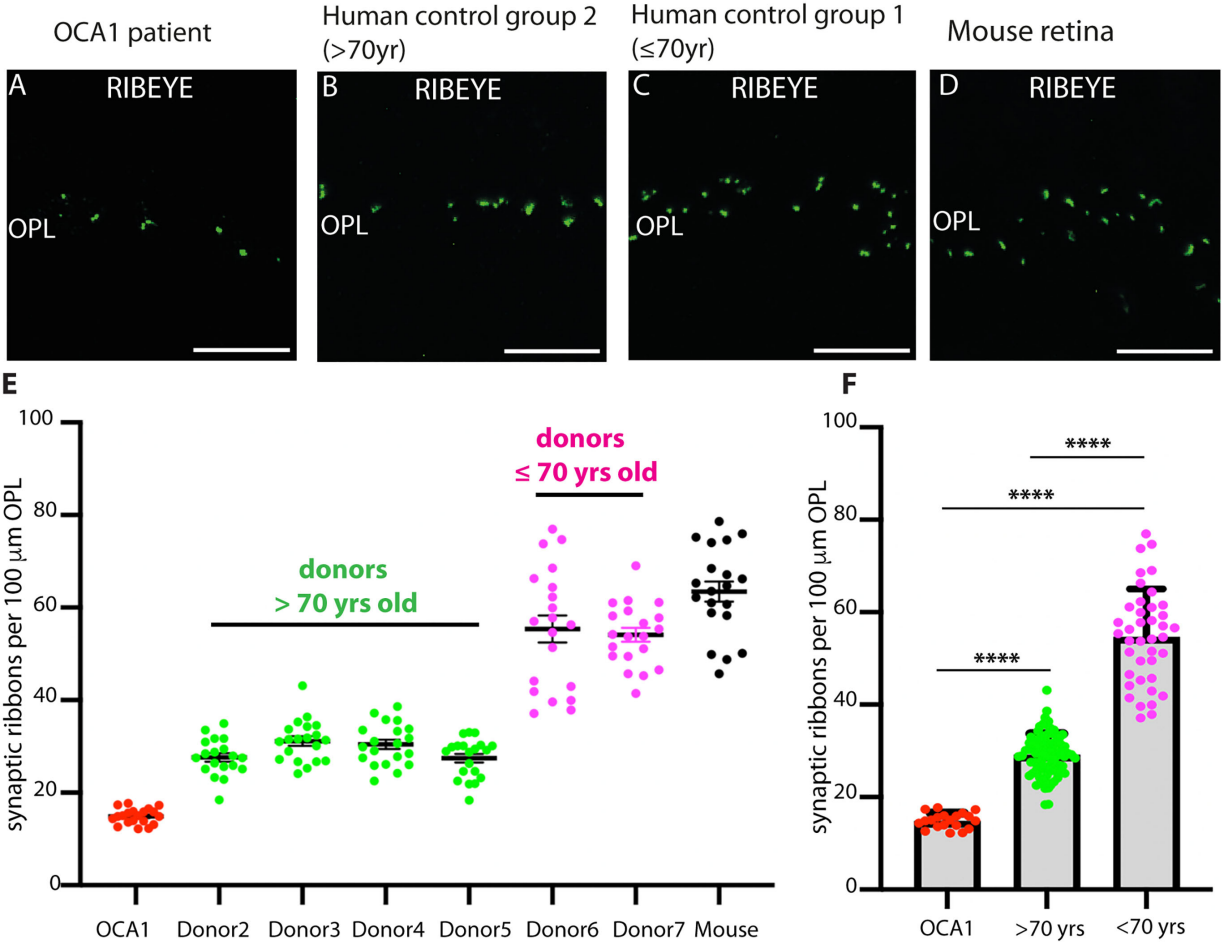
(**A–D**) Semithin sections from the indicated sources immunolabeled with antibodies against RIBEYE (**A**), the main component of synaptic ribbons. Ribbon densities in the OPL of the OCA1 patient were compared with those of donors from human controls above the age of 70 years (**B**), below the age of 70 years (**C**), and with a mouse retina sacrificed at the age of ∼7 months. (**E**) Quantification of synaptic ribbon number in the OPL normalized to the length of the OPL of the indicated individual retina samples. Statistical evaluation of differences of synaptic ribbon number per 100 µm OPL in the indicated samples. (**F**) Display of synaptic ribbon densities in the indicated pooled retina samples and statistical evaluation; only the indicated human samples are compared with each other here. ****P < 0.0001 (ANOVA with Tukey post hoc correction for multiple comparisons).

Since photoreceptor synaptic ribbons appeared to be altered not only in shape but also in number in the OCA1 patient, we quantified photoreceptor synaptic ribbon number normalized to the length of the OPL in which the photoreceptor synaptic ribbons were counted (Fig. 9). The density of synaptic ribbons in the OPL of the OCA1 patient was strongly decreased compared to control retinas (Fig. 9). We also found an age-dependent decrease of synaptic ribbons in the OPL in control human retinas (Fig. 9), similarly as previously reported for the mouse retina.^49^^,^^50^ However, the synaptic ribbon density in the OPL of the OCA1 patient was still significantly smaller than the density of synaptic ribbons from the OPL of control retinas obtained from the human donor group with the highest age (i.e., older than 70 years) (Fig. 9). Please note that, despite different postprocessing times of the samples, no significant difference in synaptic ribbon densities of photoreceptor synapses was observed within the group of eye donors older than 70 years between eye donor 2 (eye obtained from an eye enucleation with short processing time, within 1.5 hours after enucleation) and body donors 3 to 5 (eyes obtained from body donors with long postmortem and consequently delayed postprocessing time of at least 1–2 days after death), with *P* > 0.05 in ANOVA test with Tukey post hoc correction for multiple comparisons. Therefore, we assume only a limited effect of the different postmortem times on synaptic ribbon densities. This is likely based on the fact that synaptic ribbons in the retina are fairly robust, stable structures that remain intact even after prolonged isolation procedures that could last several days.^17^ Less robust structures than the synaptic ribbons could be more strongly affected by the different postmortem times.

**Figure 10.**
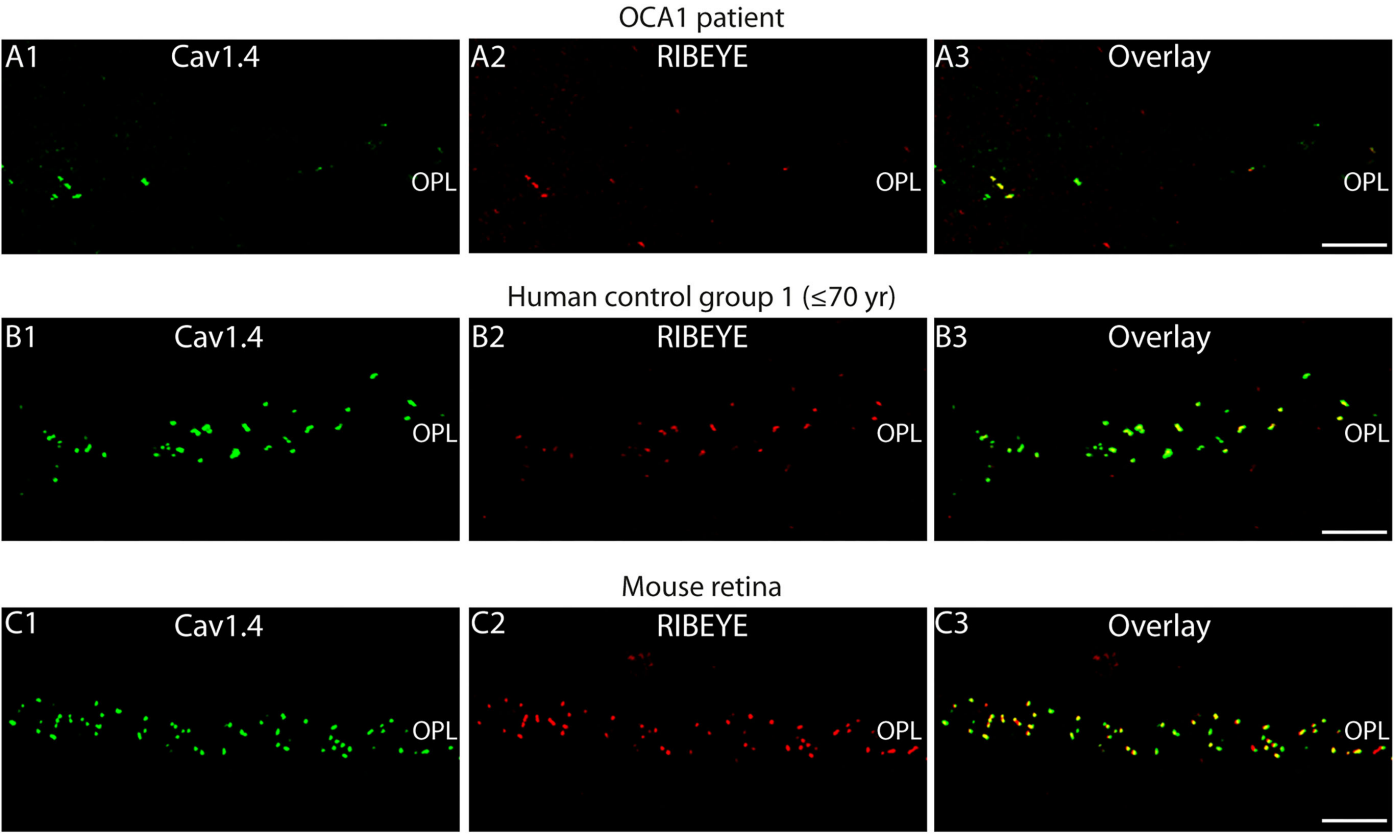
(**A–C**) Semithin sections from the indicated retinas double-immunolabeled with rabbit polyclonal antibodies against Cav1.4 (rabbit polyclonal anti-Cav1.4 Cterm) (**A1**, **B1**, **C1**, *green channel*) and mouse monoclonal antibodies against RIBEYE (clone 2D9) (**A2**, **B2**, **C2**, *red channel*). Signals are merged in **A3**, **B3**, and **C3**. *Scale bar*: 5 µm.

Double-immunolabeling of OCA1 retina and retinas from old donors (>70 years) and younger donors (≤70 years) with antibodies against RIBEYE and Cav1.4 (the voltage-gated calcium channel at the active zone that triggers exocytosis) confirmed the decrease in synaptic ribbon number in photoreceptor synapses of the OCA1 patient (Fig. 9). Interestingly, these immunolabeling experiments also showed that, whenever synaptic ribbons were found in photoreceptor synapses of the OCA1 patient, they were always accompanied by an accumulation of voltage-gated Cav-channels (Fig. 10).

This finding indicates that, whenever RIBEYE and/or synaptic ribbons were present, active zones were also assembled because voltage-gated Cav channels (Cav1.4) are physiologically highly enriched at the photoreceptor synapse active zone.^16^ In the absence of synaptic ribbons, typically no Cav1.4 clusters were observed, indicating an important role of synaptic ribbons in the organization of the photoreceptor active zone where the exocytosis of glutamatergic vesicles occurs.

We also analyzed the nerve fiber layer of the retina for morphological alterations (Supplementary Figs. S2C–S2E). The nerve fiber layer, which contains the axons of the retinal ganglion cells, looked completely normal in the OCA1 patient material at the electron microscopy level (Supplementary Fig. S2). This finding agrees with the light microscopy analysis of the OCA1 retina in Figure 1A, which did not show obvious abnormalities of the nerve fiber layer within the retina. The optic nerve of the OCA1 patient also did not show obvious alterations (Supplementary Figs. S2A, S2B).

## Discussion

In the present study, we analyzed the retina of a 35-year-old OCA1 patient with a previously diagnosed mutation in the *tyrosinase* gene.^27^ The enucleation was performed in 2018. In 2010, the patient experienced complete blindness of both eyes for largely unknown reasons. The ophthalmologists suspected a bilateral ischemic lesion of both optic nerve heads as a possible reason. However, the present data show that the optic nerve and the nerve fiber layer of the retina were intact in the OCA1 patient, as assessed by light and electron microscopy (Fig. 1, Supplementary Fig. S2). Indeed, we found a largely normal appearing retina. Only the synaptic ribbons in photoreceptor synapses were strongly altered in the OCA1 patient. They were largely absent, except for a small stump that remained associated with the active zone. These small ribbon stumps were often difficult to visualize by conventional electron microscopy because they were less electron-dense than normal synaptic ribbons. Post-embedding immunogold electron microscopy with an antibody against RIBEYE, the main synaptic ribbon protein, allowed visualization of these small stumps. The active zone-anchored synaptic ribbons in the OCA1 patient were smaller in size than in control retinas. In addition, parts of the ribbons seemed detached, fragmented, and floating in the cytosol. This observation was consistently made by light microscopy (Fig. 3C), conventional electron microscopy (Fig. 4I), and post-embedding immunogold electron microscopy (Fig. 6A). Interestingly, a previous report on albinotic mice with a defect in the *tyrosinase* gene also revealed alterations of synaptic ribbons in photoreceptor synapses in the OPL.^25^ Our present study on the retina of an OCA1 patient with a previously diagnosed *tyrosinase* gene mutation confirmed these observations made in the mouse model. In the case of the albinotic mice, the decreased size of the synaptic ribbons was found to be associated with an increased visual threshold.^25^ Thus, mutations in the *tyrosinase* gene and the resulting absence of melanin pigment in the eye affect photoreceptor synaptic ribbons in a very similar manner in the mouse and human system. In the OCA1 patient, the impact on photoreceptor synaptic ribbons appeared to be even stronger than in the albino mouse because not only was the length of synaptic ribbons affected but also the number of synaptic ribbons. This could be based on the different types and locations of the respective mutations in the *tyrosinase* gene in the albino mice and our OCA1 patient and its resulting consequences on protein structure and function.

At this juncture, the reasons why synaptic ribbons are altered in the absence of melanin synthesis in OCA1 remain unclear and hence are a subject of speculation. In the retina, melanin is mainly synthesized by the RPE, which expresses the tyrosinase enzyme under normal physiological conditions as the key enzyme for melanin pigment biosynthesis.^3^^,^^51^^–^^56^ The pigment epithelium contains large amounts of melanin in non-albinotic retinas. Photoreceptors and pigment epithelium are in close contact with each other, both topographically and at a functional level. Photoreceptors and pigment epithelium are separated from each other only by the thin subretinal space. Of note, melanin is a Ca^2+^-binding and Ca^2+^-buffering protein.^57^^–^^60^ In albinotic animals and humans, this Ca^2+^ buffering protein is absent and consequently an increased extracellular Ca^2+^ has been measured in the subretinal space of hypopigmented mice.^61^ The elevated extracellular Ca^2+^ in the subretinal space could lead to an increased Ca^2+^ influx into photoreceptors and pathologically elevated intracellular Ca^2+^ levels in photoreceptors. Increased subretinal Ca^2+^ levels could enter the photoreceptor cell via various mechanisms—for example, by increased influx through cyclic guanosine monophosphate (cGMP)*-*gated channels or voltage-gated Ca^2+^-channels.^62^^,^^63^

Pathologically elevated Ca^2^^+^ concentrations, in turn, can lead to activation of calpains and Ca^2+^-dependent intracellular proteases, as well as subsequent degradation of calpain substrates.^64^^–^^67^ Calpains might contribute to the fragmentation and degradation of synaptic ribbons we observed in the retina of the OCA1 patient. In support of this notion, calpains 3 and 1 are known to degrade CtBP1,^67^ which is closely related to RIBEYE B-domain/CtBP2.^17^ The amino acid sequences of these cleavage sites^67^ are at least partly conserved in RIBEYE B-domain/CtBP2, indicating that RIBEYE B-domain/CtBP2 may also be a substrate of calpains. Calpain 5 is particularly enriched in photoreceptor synapses,^68^ but other calpains are also present.^69^^–^^71^ Interestingly, calpain 1 has been found to be localized close to synaptic ribbons in inner ear hair cells, and its levels are increased under ototoxic conditions concomitant with a decrease in synaptic ribbon numbers and hearing loss.^72^ Further, noise-induced hearing loss is also associated with intracellular Ca^2+^ overload in inner ear hair cells, synaptic ribbon loss, and hearing loss.^73^^–^^76^ Interestingly, synaptic ribbon loss could be inhibited in this system with calpain antagonists,^76^ also suggesting a role of calpains in synaptic ribbon degradation. Finally, SNARE proteins, which execute synaptic vesicle fusion, are also calpain substrates,^77^^–^^81^ so that calpain-dependent cleavage of SNARE proteins could thus contribute to the observed visual deficits observed in the OCA1 patient we studied.

Interestingly, diseases associated with pigmentation defects other than OCA1 are also linked with synaptic ribbon alterations in photoreceptor synapses. Variants in the gene of the unconventional myosin motor protein MYO5A cause Griscelli syndrome 1 and Elejalde syndrome, which are associated with pigmentation defects and altered photoreceptor synaptic ribbons.^82^ Myosin V is expressed in both retinal pigment epithelium and photoreceptor presynaptic terminals,^82^ as well as in other presynaptic terminals of the central nervous system.^83^ Mutations of the *Rab27A* gene cause Griscelli syndrome type 2,^84^ which is also characterized by pigmentation abnormalities, including oculocutaneous albinism.^85^^–^^87^ Rab27A interacts with the active zone protein RIM2, a protein that is a highly enriched at the photoreceptor synaptic ribbon complex,^88^ and with melanophilin, MyosinVa and Munc13-4.^85^^,^^87^^,^^89^ Similarly, human Hermansky–Pudlak syndrome and its corresponding “pearl” mouse model both feature defects in pigmentation and alterations of synaptic ribbons.^90^^–^^92^ In Hermansky–Pudlak syndrome and in pearl mice, the β3A subunit of the AP3 adapter complex of vesicular trafficking is altered.^93^^,^^94^

Finally, in Waardenburg syndrome, which is associated with congenital hearing loss and abnormalities of inner ear ribbon synapses and synaptic ribbons, pigmentation abnormalities were found in the eyes and in the skin.^95^^–^^100^ The microphthalmia (Mi/MITF) transcription factors are involved in Waardenburg syndrome, and mutations in the *Mi/MITF* gene cause microphthalmic eyes and pigmentation abnormalities. The Mi/MITF transcription factors bind to an E-box-containing promotor region of the *tyrosinase* gene and E-box–containing promotor regions of other genes.^101^^–^^103^

We also observed a reduction of photoreceptor synaptic ribbons in non-albinotic retinas of aged human donors (i.e., human donors older than 70 years) (Figs. 9E, 9F). This reduction of photoreceptor synaptic ribbons in non-albinotic aged human donors was less strong than in the 35-year-old albinotic OCA1 patient. This age-dependent decrease of photoreceptor synaptic ribbons in human retinas could possibly be also related to alterations of melanin pigmentation in the RPE because the melanin contents of the RPE decreases with age.^104^^–^^108^ Decreased melanin pigmentation of the RPE is a risk factor for age-related macular degeneration (AMD).^109^^,^^110^ A photoprotective antioxidant activity of melanin in the RPE could play an important role.^111^^–^^115^ Of note, alterations of photoreceptor synaptic ribbons were observed in both human AMD patients^116^ and a mouse model of AMD.^117^ Clearly, the molecular mechanisms that link age-dependent deficiencies of pigmentation in the RPE and alterations in the presynaptic photoreceptor terminal should be elucidated by future investigations.

## Conclusions

The present study emphasizes the need to further study the molecular mechanisms that link pigmentation defects to visual system dysfunctions and synapse alterations in the visual pathway with respect to the development of novel therapeutic strategies for patients that suffer from genetic pigmentation diseases, as well as for photoreceptor synapse dysfunctions caused by age-related melanin pigment dysfunctions in the RPE.

### Limitations of the Study

To improve comparability between samples, the human retina pieces analyzed in the study were dissected from a central portion of the human retina located between the macula and the optic nerve in the nasal region of the retina. Within that area we did not further consider potential regional differences. Thus, within this area we cannot exclude potential local morphological differences and possible variations between the dissected human retina samples. It also should be emphasized that we focused on human tissue in the present study in which we analyzed retinas from human donors with an individual patient history. Due to the ethical and legal regulations and practical limitations in the availability of human eye donors, the age of the available human eye donors had a wide range. Based on these ethical, legal, and practical limitations, it was not possible to match the age of the retina from the 35-year-old albinotic OCA1 patient with a retina from a non-albinotic eye donor of the same age, as a retina from a non-albinotic donor at the same age of 35 years was not available to us for analysis. Furthermore, it should be noted that only a single OCA1 patient with the described specific mutations in the *tyrosinase* gene was analyzed in the present study. Future studies of additional human OCA1 patients might be encouraged by the present study.

## Supplementary Material

Supplement 1
