# Supplementary material for: Alterations of Photoreceptor Synaptic Ribbons in the Retina of a Human Patient With Oculocutaneous Albinism Type 1 (OCA1)
Source: Invest Ophthalmol Vis Sci. 2025 Oct 8;66(13):14. doi: 10.1167/iovs.66.13.14 (PMC12517378; doi:10.1167/iovs.66.13.14)
Supplement: Supplement 1 [file iovs-66-13-14_s001.pdf]

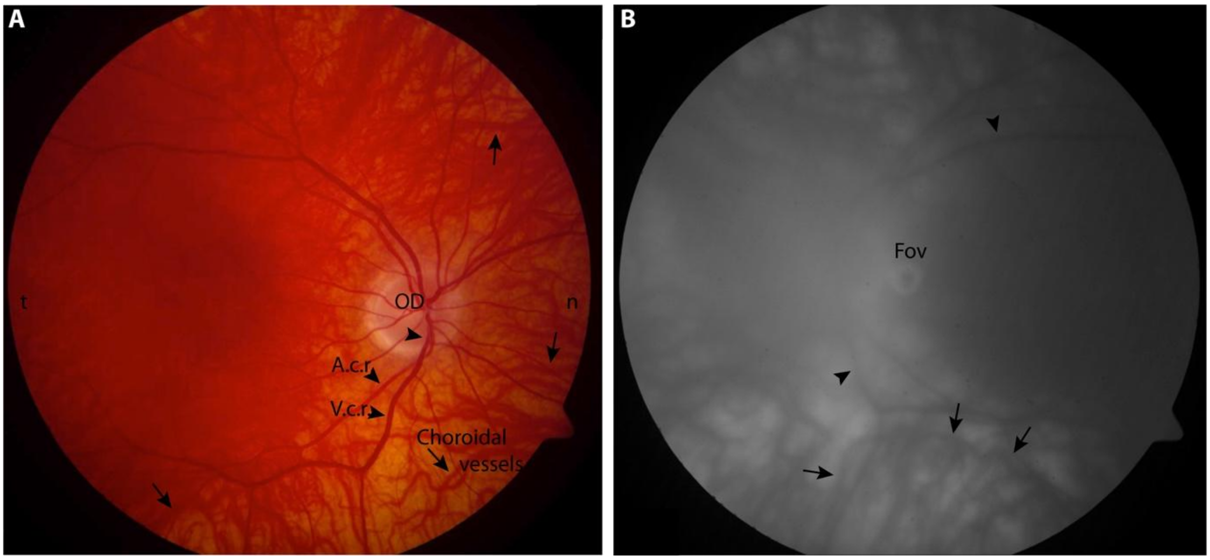

Supplemental Figure 1. *(A,B) Ophthalmoscopic fundus image of the OCA1 patient (recorded in 2011, as the OCA1 patient was 28 years old). Due to the absence of melanin pigment in the RPE not only the retinal vessels (central retinal artery / vein with their branches; denoted by arrowheads) are visible but also the choroidal vessels (short posterior ciliary arteries/veins and their branches; denoted by arrows). (A) shows the entire fundus; (B) shows a higher magnification of the macula region. Abbreviations: OD, optic disc; t, temporal; n, nasal; A.c.r., central retinal artery; V.c.r., central retinal vein; RPE, retinal pigment epithelium; Fov, Fovea.*

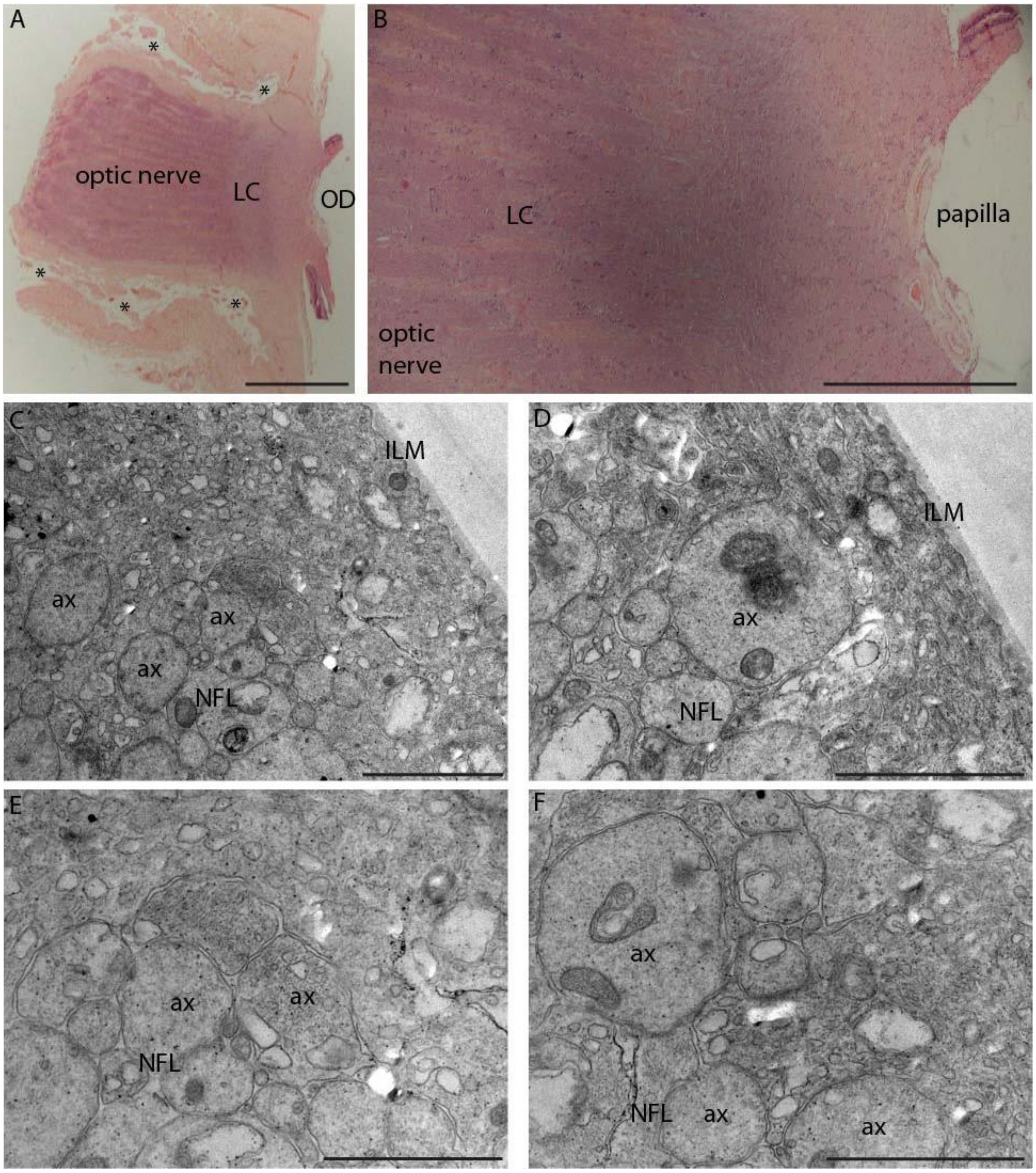

Supplemental Figure 2. *(A,B) HE-stained paraffin section of the optic nerve of the OCA1 patient as it leaves the eye-cup. (C-F) transmission electron microscopy of the inner retina from the OCA1 patient showing unmyelinated axons from retinal ganglion cells in the nerve fiber layer (NFL). Abbreviations: LC, Lamina cribrosa; ILM, inner limiting membrane; OD, optic disc; NFL, nerve fiber layer; ax, unmyelinated axon of retinal ganglion cells; asterisks denote subarachnoid space. Scale bars: 1 mm (A,B), 2 μm (C); 1 μm (D-F).*
